# The Book World of Medicine and Science

**Published:** 1905-12-30

**Authors:** 


### The Book World of Medicine and Science.

Medicine and the Public. By S. Squire Sprigge, M.A., M.D. (London : William Heinemann. 1905. Price 6s.)

The author published as a series of articles in the *Lancet* many of the chapters in this book; by bringing them out now in book form he hopes that the various reforms in the medical profession that he advocates may reach the lay public more effectually than through the columns of a medical paper like the *Lancet*. In the chapter on hospital abuse Dr. Sprigge shows convincingly what a poor place the municipal or State supported general hospital would be; and yet it is to this climax that we seem to be gradually tending. The labouring man pays his penny a week towards some hospital charity, and then expects and demands free treatment whenever he is ill; he expects, too, to have some control in the management of the institution, and from this it is only a step to the municipalisation of the hospital. The article that deals with quackery shows how much legislation is required for the benefit of both the profession and the general public. Several chapters are devoted to the subject of medical education, and how it could be improved. The preliminary examination, before entering on the five years' medical course, might be made more searching: in this way "the student who is bound to extend a five years' professional education into one of seven, or even more, years, because his general education has been neglected, would be barred at the outset, instead of being allowed to struggle on, passing his subjects piecemeal and attacking each stage of his career with all the disabilities attending an imperfect knowledge of what he has put behind him. To turn out practical medical men is, or should be, the aim of medical education. This result is not achieved by admitting all and every to the professional examinations." The habit of multiplying examinations and of splitting the natural grouping of the subjects of the curriculum into their component parts, each of which the student may take separately, is most prejudicial to the attainment of a thorough practical knowledge of medicine; it leads to the student spending his time learning by rote things that he believes will secure him a safe passage through the next examination. The reforms advocated in this book are moderate, and their necessity is convincingly insisted upon. The following are some that are suggested and discussed:—The prohibition of unqualified medical practice; the institution of one State examinaticn, by which the medical profession must be entered; an imperial sanitary service which would include the Army, Navy, Indian, and Colonial Medical Services; a Ministry of Public Health. With regard to the last, the following are the author's words:—"As the public becomes better educated, as it recognises how much depends upon the observance of laws of public health, we may expect a general consensus of opinion as to the advisability of a Ministry of Public Health, the officers of which, properly selected for a special knowledge in preventive medicine, properly paid, and free from any hampering conditions (whose tenure of office is not dependent upon the goodwill of some ignorant or self-interested Municipal Council), may safeguard the health of the country and watch over the interests of the sick poor." This book should most certainly be read by those interested in the welfare and progress of the community as a whole, for Dr. Sprigge clearly shows that the reforms which he advocates will at the same time further both the best interests of the profession and those of the general public. To the medical man the book is full of terse statements of facts with which he is well acquainted, and for the most part fully in accord; would that the lay public might take the trouble to study them, too, and interest itself in medicine from day to day, and not only on occasions when great emergencies arise.

The Physiology and Therapeutics of the Harrogate Waters, Baths, and Climate Applied to the Treatment of Chronic Disease. By William Bain, M.D., M.R.C.P., and Wilfrid Edgecombe, M.D., F.R.C.S. (London: Longmans, Green and Company. 1905. Pp. 300. Price 7s. 6d. net).

Works belonging to this class are usually considered, and not unjustly, as legitimate means for securing benefit to the author or the advertisement of the health resort described. The present work, however, is much more than a mere spa manual. It is written by experienced clinicians imbued with the scientific spirit. In form and substance the work is excellent. The material, which has evidently been collected with painstaking care and selected with judicious discernment, is arranged in four sections—dealing respectively with the waters, baths, and climate of Harrogate, and their employment in various chronic affections. In each case an attempt has been made to indicate the general principles involved, the nature of the physiological action, and the therapeutic application. The work is well designed and has been faithfully carried out. Every practitioner before advising resort to Harrogate should read this clearly written and highly instructive description of one of the most popular and serviceable of our British health stations. The work is well printed on particularly light paper and it is thus a pleasure both to read and to handle this suggestive and informing volume.

Lunacy Practice. A Practical Guide for the Certification and Detention of Persons of Unsound Mind. By William Gattie, F.C.I.S. (London: Simpkin, Marshall and Company. Pp. 53. 2s. 6d. net.)

Unquestionably this book supplies a want. It may be true that in some medical jurisprudence classes the students are instructed in the proper way to fill up lunacy certificates, and an outline of the law on the subject may be included in the course of lectures; but in the hurry of general practice these lessons are apt to be forgotten, and the practitioner is confronted with his ignorance only when the time comes that he has to certify to the insanity of his patient. Everyone connected with an asylum knows how often, how very often, reception orders are faulty, and although the errors may not amount to invalidity, they entail a great deal of trouble and correspondence between the asylum clerk, the practitioner, and the Commissioners in Lunacy. In addition to the mere certification of patients, the little volume contains much information relative to the treatment of the insane as single patients in private care, and the formalities necessary to be observed for their reception and detention. As regards the examination of supposed lunatics, the author lays stress on the importance of detecting a distinct delusion; but delusion is not always present, and when present it is not always easy to detect. We believe that if the practitioner will carefully examine his patient under the three heads of how he looks, what he does, and what he says, he will not often have much difficulty, providing the patient is insane, in finding definite evidence of insanity which may be placed in the certificate. Every medical man would do well to provide himself with Mr. Gattie's useful book, and to consult it when he is in doubt of the routine to be followed.

